# Topical Nitroglycerine for Neonatal Arterial Associated Peripheral Ischemia following Cannulation: A Case Report and Comprehensive Literature Review

**DOI:** 10.1155/2013/608516

**Published:** 2013-10-23

**Authors:** Rafat Mosalli, Mohamed Elbaz, Bosco Paes

**Affiliations:** ^1^Department of Pediatrics, Umm Al Qura University, P.O. Box 7607, Mecca 21955, Saudi Arabia; ^2^Department of Pediatrics, International Medical Center, Hael Street, P.O. Box 2172, Jeddah 21451, Saudi Arabia; ^3^Department of Pediatrics (Neonatal Division), McMaster University, HSC-3A, 1280 Main Street West, Hamilton, ON, Canada L8S 4K1

## Abstract

Arterial cannulation in neonates is usually performed for frequent blood pressure monitoring and blood sampling. The procedure, while easily executed by skilled neonatal staff, can be associated with serious complications such as vasospasm, thrombosis, embolism, hematoma, infection, peripheral nerve damage, ischemia, and tissue necrosis. Several treatment options are available to reverse vascular induced ischemia and tissue damage. Applied interventions depend on the extent of tissue involvement and whether the condition is progressive and deemed life threatening. Standard, noninvasive measures include immediate catheter removal, limb elevation, and warming the contralateral extremity. Topical vasodilators, anticoagulation, thrombolysis, and surgery are considered secondary therapeutic strategies. A comprehensive literature search indicates that topical nitroglycerin has been utilized for the treatment of tissue ischemia in three preterms with umbilical arterial catheters and four with peripheral arterial lines. We report the first successful use of nitroglycerine ointment in a critically ill preterm infant with ischemic hand changes after brachial artery cannulation.

## 1. Introduction

Arterial catheter insertion in the premature infant is usually undertaken to facilitate easy blood sampling for required diagnostic investigations and to monitor blood pressure and hemodynamic stability in a quiescent state. This is normally accomplished through the insertion of indwelling umbilical arterial catheters soon after birth or, if unsuccessful, the alternative placement of peripheral arterial lines in common sites such as the radial, ulnar, dorsalis pedis, or posterior tibial arteries. Prior to initiation of the procedure, circulatory sufficiency, evidence of adequate collateral blood flow at the selected site, absence of bleeding diatheses, local skin infection, and limb malformation are basic prerequisites [1]. Preterm neonates have a small arterial diameter relative to the catheter size, and they are therefore more susceptible to complications such as iatrogenic trauma, vasopasm, thrombosis, and thromboembolism with subsequent tissue ischemia and necrosis of the involved anatomical region.

The usual treatment of ischemic injuries includes immediate removal of the catheter, elevation of the affected limb, and application of warm compresses to the opposite limb (reflex vasodilation), but these maneuvers afford variable success. Anticoagulants such as unfractionated [2, 3] or low molecular weight heparin [2, 4, 5] and thrombolysis with tissue plasminogen activator [6–9] are primarily employed when complete thrombotic occlusion of the vessel occurs with rapidly progressive ischemia, and the risks associated with surgery are considered substantial or even detrimental [2]. Streptokinase and urokinase [10] have been utilized in case reports, but overall none of the drug strategies have been evaluated through robust, controlled clinical trials.

A comprehensive literature search was conducted of existing scientific databases, and 7 case reports of peripheral ischemia due to arterial cannulation in the neonate were identified with complete recovery attributed to the application of topical nitroglycerine. We describe the first case which involved catheter insertion into the brachial artery.

## 2. Case Report

A Saudi Arabian girl weighing 520 g was born at 25 weeks gestational age to a 35-year-old, gravida 4, para 3 mother. There was no family or medical history of a thrombophilic disorder. The infant was a vaginal delivery with Apgar scores of 3 at 1 minute and 8 at 5 minutes. The birth weight was appropriate for gestational age with no dysmorphic features. The infant was immediately intubated, received surfactant, and was mechanically ventilated. The initial chest X-ray was compatible with respiratory distress syndrome. Shortly after birth, the baby developed hypotension. Umbilical venous and arterial lines were inserted, and a dopamine drip was started at 10 mcg/kg/minute with subsequent stability. A partial sepsis workup was performed, and the baby was started on ampicillin and gentamicin. Hyperglycemia on day 2 was managed with an insulin infusion. Cerebral ultrasound on day 3 of life was normal. The umbilical venous and arterial lines were removed on day 7 of life.

**Figure 1:**
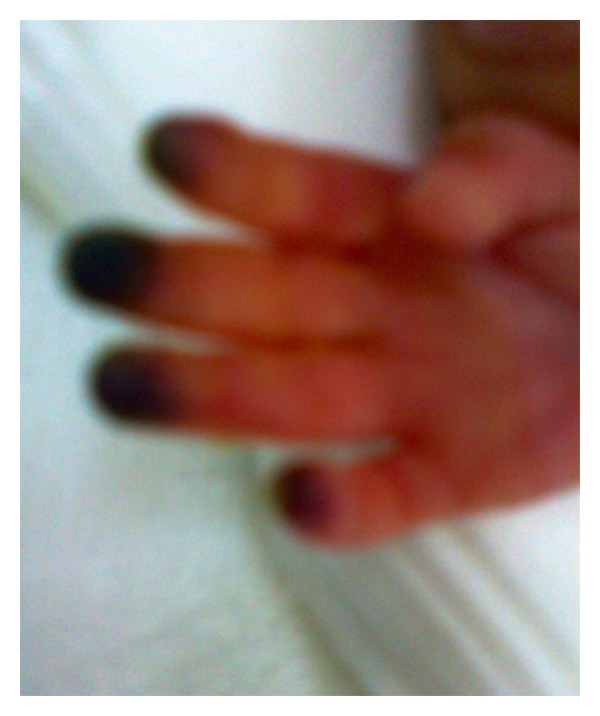
Digital involvement of the right hand 8 hours after brachial artery cannulation.

**Figure 2:**
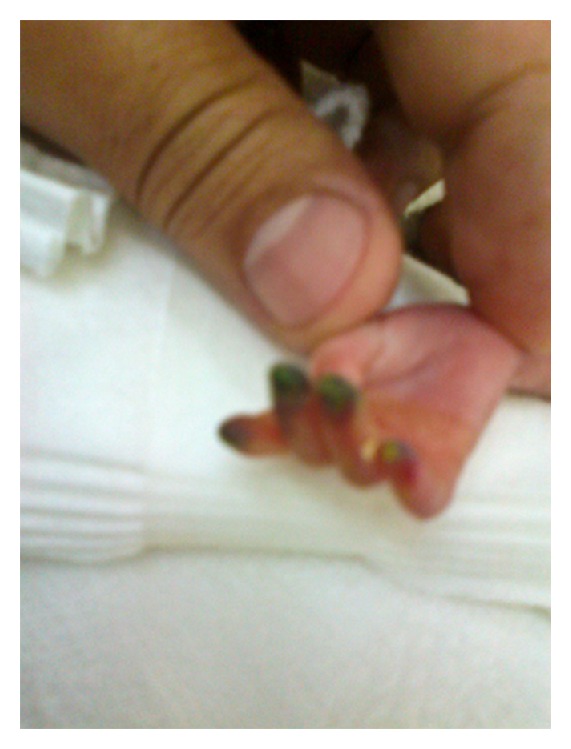
Signs of early necrosis in the finger tips.

On day 14, the baby developed acute renal failure secondary to sepsis with poor urine output, hypotension, metabolic acidosis, hyperkalemia, and a raised creatinine. Numerous attempts were made to insert a peripheral arterial line for frequent blood sampling and blood pressure monitoring without success. Due to the severity of illness and need for arterial access, a decision was made to insert a right brachial artery catheter, but after 8 hours the middle, ring, and index fingers of the right hand were noted to be cold, and cyanosed, and the arterial line was immediately removed (Figure 1). The hand was elevated, and warm compresses were applied to the contralateral limb. Ischemic changes soon became evident and 4 hours later progressed to involve the distal part of the fingers which started showing signs of probable early necrosis (Figure 2). The international normalized ratio (INR) and partial thromboplastin time were normal. A complete prothrombotic screen was not performed because the event was not spontaneous, there was no family history of thrombophilia, and there was a causal relationship of the ischemia with the brachial cannulation procedure [11]. A Doppler ultrasound indicated a sluggish flow through the brachial artery without definite visualization of a thrombus which suggested that the underlying etiology was more likely vasospasm rather than thrombosis. Anticoagulation was considered as one possible strategy to preserve perfusion and prevent digital loss, but the risks of heparinization and potential bleeding were considered significant. A ribbon of 2% nitroglycerin ointment (less than 4 mm/kg) was initially sparingly applied to the fingers, approximately 2 cm proximal to the line of pallor. Slight improvement in color and perfusion was noted in the fingers over the next 8 hours. Topical nitroglycerin was subsequently applied every 8 hours, 1 cm proximal to the ischemic site, following the anatomic course of the brachial artery. Methemoglobin levels were monitored daily and stayed in the normal range (metHgb < 1%).

**Figure 3:**
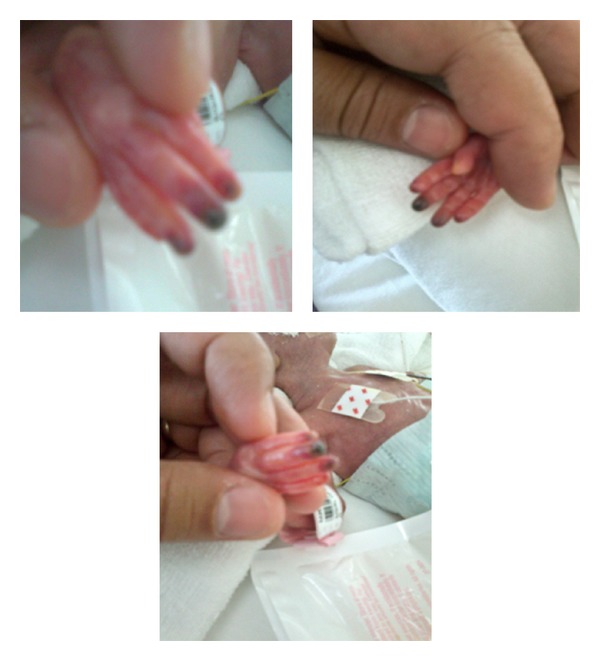
Resolving ischemia of the 2nd, 3rd, 4th and 5th fingers, 5 days after the ischemic event (dorsal and palmar aspects of the hand).

**Figure 4:**
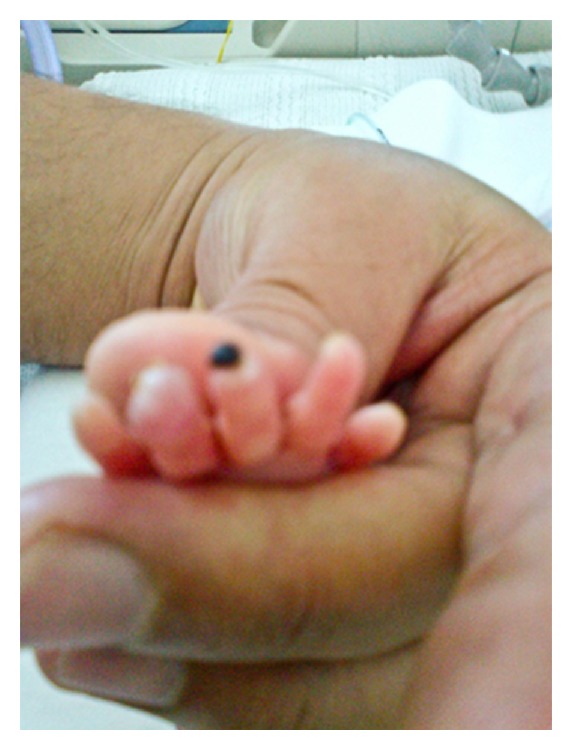
Residual ischemia at the tip of the middle finger after 10 days.

**Figure 5:**
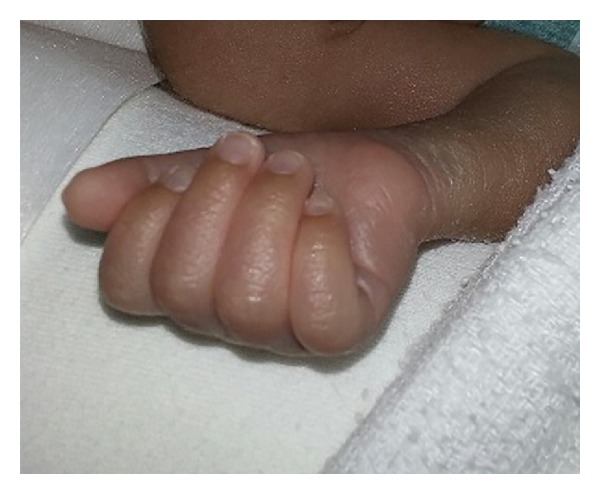
Complete resolution 7 weeks after the incident.

There was a gradual improvement in color and capillary refill over the next several days. By day 12, the area of ischemia was limited to the tips of the fingers, and treatment was discontinued on day 21 (Figures 3 and 4). The nail beds were intact with full restorative function of the fingers (Figure 5). The baby made an uneventful recovery from her renal failure, was extubated at 1 month of age, and was eventually discharged home at 34 weeks corrected gestational age, weighing 1.85 kg.

## 3. Discussion

Neonates have several risk factors that predispose them to thrombosis and catheter induced ischemia [11]. A systematic review of spontaneous neonatal arterial thromboembolism classified these as congenital, acquired, inherited prothrombotic abnormalities, and maternal [12] factors. The coagulation system in newborns matures slowly with mean plasma concentrations of the vitamin-K dependent factors, contact factors, and direct inhibitors of thrombin being approximately 50% of adult values at birth [13–15], whereas *α*2-macroglobulin, factors V, VIII, and XIII, and von Willebrand factor are increased in the first few weeks of life [16, 17]. Healthy preterms gradually attain adult coagulant protein levels by 6 months of age, but in the interim any illness may disrupt hemostasis leading to a fall in *α*2-macroglobulin, with subsequent thrombosis and ischemia [18]. Moreover, the placement of catheters may cause additive vascular endothelial damage which incites a rapid inflammatory cascade that enhances platelet adhesion and aggregation through the release of adenosine diphosphate and thromboxane, resulting in localized stasis, reduction in blood flow, and thrombosis [19]. The affected area is soon compromised with absent pulses and becomes pale, cool, and markedly discolored [20].

There are recommended sites for central and peripheral arterial vascular access which are considered relatively safe because of a collateral circulation (radial, dorsalis pedis, posterior tibial arteries, and less commonly ulnar) [21, 22]. The respective vessels are also easily identified with a fibreoptic source, or the vessels are directly visible for catheterization (umbilical arteries). However, these purported safe sites are not without risk from ischemic changes and digital or limb necrosis [23–26]. Equally, the placement and position of umbilical arterial catheters may result in clinical vascular compromise. In a Cochrane meta-analysis of 5 randomised or quasirandomised studies, Barrington [27] reported that ischemic phenomena were significantly less common with high positioned catheters (tip in the descending aorta, above the level of the diaphragm and below the subclavian artery; RR 0.53, 95% CI 0.44–0.63). The author recommends that the practice of low placed umbilical catheters should be abandoned because of the inherent risks.

Brachial artery cannulation or puncture for blood sampling should be avoided in infants because of the risk of thrombosis and proximity to the median nerve, unless the indication is urgent and access is no longer available through the usual sites [28]. However, Schindler et al. in a retrospective study, described their safe experience with 112 brachial arterial lines in infants <5 kg [29]. Reported complications were few: temporary occlusion (*n* = 1), local infection (*n* = 1), and local hematoma (*n* = 5). There are several reports of significant complications with brachial artery catheterization. Giaquinta et al. described a 3-day-old infant, who sustained major ischemic changes following an attempt to cannulate the brachial artery [30]. Microvascular, reconstructive surgery was performed to salvage blood flow to the limb. In our case, similar problems ensued, and a trial of topical nitroglycerin proved successful. Coombs et al. described a series of eleven ill patients aged 1 day through 2 years, four of whom had a gestational age between 26 and 31 weeks and a birth weight range of 980 to 2200 g [31]. The infants had repeated radial and brachial artery punctures in an attempt to establish vascular access which resulted in vessel occlusion and severely compromised limb perfusion. All underwent arteriotomy, embolectomy, and reconstructive vascular surgery with good outcomes. The authors developed an algorithmic, multidisciplinary approach to brachial artery thrombosis which weighs the risk of benefit versus harm for each instituted therapeutic intervention, while mandating surgery for absolute indications.

Nitroglycerine is a known smooth muscle relaxant which is readily absorbed through intact skin. Preterm infants have an immature epidermal permeability barrier which likely enhances drug absorption and may produce systemic complications [32, 33]. Nitroglycerine forms free radical nitric oxide which activates guanylate cyclase and increases cellular guanosine 3′, 5′-cyclic monophosphate. This dephosphorylates smooth muscle myosin which regulates the contractile state and results in vasodilation [34]. The amount of absorbed nitroglycerine and systemic levels achieved is likely directly related to the quantity of ointment and size of the area of application [35]. The usual starting dose of 2% nitroglycerine ointment is 4 mm/kg (or 2 mg measured as 0.1 mL in a syringe), which is equivalent to 0.2–0.5 *μ*g/kg/min administered intravenously [36]. The vasodilation is evident within 15–30 minutes, and the hemodynamic effects may be sustained for as long as 6 hrs.  Side effects can occur such as hypotension, tachycardia, flushing, and methemoglobinemia due to nitric oxide production. Hypotension and tachycardia were documented in a few reports [37–39] but resolved in the majority of cases without treatment. Prolonged use of nitroglycerine may cause methemoglobinemia, and monitoring of levels is important. However, this complication has only been noted in adults receiving high doses of intravenous nitroglycerin [40, 41].

Topical nitroglycerin has been used in neonates for several indications. A search of MEDLINE, PubMed, Web of Science, CINAHL, Cochrane Databases, DARE, and OVID was performed using the following mesh terms: nitroglycerin OR topical nitroglycerin AND artery OR arterial catheter AND tissue ischemia OR necrosis AND infant-newborn OR neonate. Retrieved citations were also scanned for additional pertinent references. The search yielded 14 articles, six of which were relevant. Two articles reported 3 cases of ischemia: one in association with a femoral artery cannulation in a 31-week premature infant at 2 months of age [6], one with a left axillary artery injury during central venous catheter placement [42], and one case during radial artery sampling [42]. In the latter two examples, complete recovery occurred with treatment while, in the former, aggressive management with tissue plasminogen activator, heparin, and topical nitroglycerin resulted in a relatively good outcome with residual dry gangrene at the tip of the left great toe. Topical nitroglycerin has been employed to aid in the insertion of peripheral venous catheters in neonates, but use was discontinued because of limited success and accompanying concerns of hypotension which may have been dose-related [35, 43, 44]. Nitroglycerin ointment may also be beneficial in tissue ischemia following extravasation of parenteral solutions [45]. Currently, there are 4 published studies on the use of topical nitroglycerin in neonates for the reversal of peripheral ischemia due to arterial cannulation. These involve 7 preterm infants (gestational age range 23–33 weeks), and the outcomes are uniformly positive (Table 1) [37–39, 46]. Our report in which repeated doses of topical nitroglycerin relieved distal ischemia and resulted in complete resolution and normal perfusion of the affected hand adds to the existing case series. However, the underlying pathophysiological mechanism suggests that recovery may only occur in cases associated with intense vasospasm of the traumatized vessel and is perhaps less likely to resolve with true embolism or occlusive thrombosis without additional anticoagulation and thrombolysis.

In essence, anticoagulation with unfractionated heparin should be reserved for definite occlusive thrombi. Low molecular weight heparin may also be used if no invasive procedures or thrombolytic therapy is planned [2, 12]. Thrombolytic therapy with tissue plasminogen activator should be instituted for thrombi unresponsive to unfractionated heparin in the presence of progressive tissue ischemia. Absolute indications for surgery include total limb ischemia, evidence of compartment syndrome or pregangrenous tissue evolution, and documented absence of arterial blood flow by Doppler ultrasound or angiography for >24 hours [12, 31].

The only other reported therapeutic option for neonatal limb ischemia associated with arterial vasopasm is sympathetic blockade. The anesthetic procedure directly induces vascular dilation through a mechanism that is not endothelium dependent [47] and is conjointly enacted through the inhibition of afferent impulses that reduces pain-related release of epinephrine and norepinephrine and decreases peripheral vasoconstriction [48]. De Carolis et al. [49] describe a series of 12 cases in which peripheral nerve blockade alone (*n* = 6) or combined with anticoagulation (*n* = 4) was safely employed to treat limb ischemia. Thrombosis was reported occurring soon after birth (*n* = 2) or in association with umbilical arterial catheterization (*n* = 8), peripheral arterial puncture (*n* = 1), or cannulation (*n* = 1). However, in the described cases complete recovery occurred in 5 infants, 5 required subsequent amputations of a limb or digits and one died. More recently, Ponde et al. [50] further documented residual ischemia of the finger tips following an infraclavicular brachial plexus block in a 900 g, female infant who had an accidental peripheral arterial puncture. The authors [49, 50] recommend peripheral nerve blockade as an effective procedure for arterial vasospasm, but the outcomes indicate significant morbidity and stress the importance of a multidisciplinary approach to limb ischemia as advocated by Coombs et al. [31].

## 4. Conclusion

Topical 2% nitroglycerin ointment at a dose of 4 mm/kg may be a useful initial therapy for the reversal of tissue ischemia in premature and term babies who do not respond to standard noninvasive measures after peripheral arterial cannulation. Several questions remain unanswered. The optimum time to commence treatment after the ischemic event, the frequency and safety of administration in newborns especially preterm infants, and the use of nitroglycerin in combination with other therapies has not been established. Therefore, prospective studies evaluating specific prescriptive regimens are necessary before this therapeutic option is implemented in clinical practice for arterial vasospasm. Anticoagulation and thrombolysis should be considered for progressive ischemia but should never be a substitute for surgery. The approach to an infant with limb ischemia is critical and needs to be systematic, collaborative, multidisciplinary, and standardized in order to affect the best short- and long-term outcomes. It is important to note that if topical nitroglycerin therapy is instituted, consultation should be concurrently sought from hematology and surgery to facilitate timely intervention and avoidance of vascular compromise.

## Figures and Tables

**Table 1:** Literature review of neonates treated with nitroglycerin for arterial catheter-related ischemia and their outcomes.

Author/Year [Reference]	Gestational age, wk; (Birth wt, g)	Catheter type	Region and clinical findings	2% nitroglycerin topical dose	Outcome
Wong et al./1992 [37]	25; (700), 24; (780)	(1) Right radial (2) Right radial	(1) No pulse, right hand blanched (2) no pulse, blue fingers, right hand rigid	Ribbon 4 mm/kg in both cases	(1) Improvement within 15 min, full recovery in 3 hr (2) Two doses with complete recovery after 16 hr except for 2 small blue areas.

Varughese and Koh/2001 [38]	33; (1870)	UAC	Ischemic changes over right hip. Normal femoral + popliteal pulses	0.4 mg over 2 hr—twice	Improvement over 7 hr with complete resolution after 30 hr

Baserga et al./2002 [39]	(1) 30; (1620) (2) 25; (not stated) (3) 23; (660)	(1) UAC (2) UAC (3) Left radial	(1) Poor perfusion left leg, weak femoral + tibial pulses (2) Right leg blanched, absent femoral pulse, cyanotic toes (3) Pale left hand and fingers, absent radial pulse	(1) 4 single applications over affected parts-dose not stated (2) 4 mm/kg—four single applications over affected parts (3) ribbon of ointment-dose not stated-3 single applications to affected areas	(1) Complete recovery in 45 min (2) Complete recovery in 45 min (3) Improvement in 30 min with full recovery.

Vasquez et al./2003 [46]	26; (896)	Left peripheral arterial line (location not stated)	Pale cyanotic left hand with discoloration from mid-palm to finger tips.	Ribbon 4 mm/kg q 8 hr for 27 days	Improvement in 8 hr with gradual recovery over 18–27 days. No deficit at 8 months.

UAC: umbilical arterial catheter.
